# On the Employment of Hairpowder in Erysipelas

**Published:** 1832-09

**Authors:** 


### HAIRPOWDER IN ERYSIPELAS.

### *On the Employment of Hairpowder in Erysipelas.*

*To the Editors of the London Medical and Physical Journal.*

GENTLEMEN: A few months since, being in attendance on a medical friend, who was confined to his bed by erysipelas, I recommended the application of hairpowder. He said he had no faith in it, as he could not understand how merely sprinkling hairpowder on the skin was to diminish inflammation. I said in reply, how does covering the polished surface of a metallic vessel, containing hot water, render the cooling process more rapid? And, by the way, I added, as the thought of the moment, covering an inflamed surface with hairpowder may act in the same manner; namely, *it may increase the radiation of heat*, and thus be tantamount to an *evaporating* lotion, without its danger.

It was proposed to ascertain this point by experiment, which would be a matter of no great difficulty; but for this I have since had neither leisure nor inclination. The practice of sprinkling hairpowder on the skin affected with erysipelas is both old and common, and I have heard the *cooling* effect of it described as extremely grateful; but I am not aware that any rational explanation of its *modus operandi* has been suggested. If this should be the true principle of its operation, (which remains to be ascertained,) the application of it may perhaps be advantageously extended.

I am, Gentlemen, your obedient servant,

L. G.

*Bath; July 9th,* 1832.

